# The topological Anderson insulator phase in the Kane-Mele model

**DOI:** 10.1038/srep24007

**Published:** 2016-04-05

**Authors:** Christoph P. Orth, Tibor Sekera, Christoph Bruder, Thomas L. Schmidt

**Affiliations:** 1 Department of Physics, University of Basel, Klingelbergstrasse 82, 4056 Basel, Switzerland; 2 Physics and Materials Science Research Unit, University of Luxembourg, L-1511 Luxembourg.

## Abstract

It has been proposed that adding disorder to a topologically trivial mercury telluride/cadmium telluride (HgTe/CdTe) quantum well can induce a transition to a topologically nontrivial state. The resulting state was termed topological Anderson insulator and was found in computer simulations of the Bernevig-Hughes-Zhang model. Here, we show that the topological Anderson insulator is a more universal phenomenon and also appears in the Kane-Mele model of topological insulators on a honeycomb lattice. We numerically investigate the interplay of the relevant parameters, and establish the parameter range in which the topological Anderson insulator exists. A staggered sublattice potential turns out to be a necessary condition for the transition to the topological Anderson insulator. For weak enough disorder, a calculation based on the lowest-order Born approximation reproduces quantitatively the numerical data. Our results thus considerably increase the number of candidate materials for the topological Anderson insulator phase.

Topological insulators (TIs) are novel materials which have raised a great deal of interest over the past decade^1^,^2^. One of their distinguishing features is the existence of conducting boundary states together with an insulating bulk. The boundary states are protected by time-reversal symmetry (TRS) and exist both in two-dimensional (2D) and three-dimensional (3D) TIs. In 2D TIs, the boundary states lead to an edge conductance of one conductance quantum per edge for chemical potentials inside the bulk band gap^3^,^4^,^5^.

It is a challenging task to find candidate materials for TIs. So far, only a limited number of materials are known. The most prominent 2D TIs are HgTe/(Hg, Cd)Te quantum wells (HgTeQWs)^6^ and InAs/GaSb heterostructures^7^,^8^, whereas 3D TIs were found for instance in Bi_1−*x*_Sb_*x*_^9^. The fact that their metallic surface states emerge due to a topological property of the bulk band structure means that they are robust to weak disorder. However, one expects that a large amount of disorder should ultimately localize the surface states and render them insulating.

All the more surprising, it was predicted that the opposite transition can happen in certain parameter ranges: adding strong disorder can convert a trivial insulator without edge states into a topological insulator with perfectly conducting edge states. Materials that exhibit this new state have been termed topological Anderson insulators (TAIs).

This effect was first theoretically predicted based on the lattice version of the Bernevig-Hughes-Zhang (BHZ) model for HgTeQWs in the presence of Anderson disorder^10^,^11^. For Anderson disorder, a random on-site potential, uniformly distributed in an energy window of width 2*W*, is assigned to each lattice site of a tight-binding model. From Anderson’s theory of localization^12^ one expects that a system with finite conductance without disorder undergoes a transition to a system with localized states and suppressed conductance as the disorder is increased beyond a certain threshold value. The behavior of TAIs instead is quite different. A TAI is an ordinary band insulator in the clean limit. Above a critical disorder strength *W*, an interesting topological state appears, in which the material features a quantized conductance. For even stronger *W*, above the disorder strength at which the states of the conduction and valence band localize, it was proposed that tunneling across the bulk becomes possible^13^, probably enabled by percolating states^14^, and the conductance is again suppressed.

The disorder-induced transition can be understood by a renormalization of the model parameters. The BHZ model with disorder and band mass *m* can be approximated by an effective model of a clean system and renormalized mass 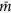. Using an effective-medium theory and the self-consistent Born approximation (SCBA), it was shown that for certain model parameters, 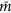 can become negative even if the bare mass *m* is positive^15^. As a consequence, the effective model becomes that of a TI and features edge states with a quantized conductance of *G*_0_ = *e*^2^/*h*^16^.

Furthermore, TAIs have been predicted in several related systems, for instance in a honeycomb lattice described by the time-reversal-symmetry breaking Haldane model^17^, a modified Dirac model^17^, the BHZ model with *s*_*z*_ non-conserving spin-orbit coupling^18^, as well as in 3D topological insulators^19^. Moreover, similar transitions from a topologically trivial to a topologically nontrivial phase have been found to be generated by periodically varying potentials^20^ or phonons^21^. In contrast to on-site Anderson disorder, certain kinds of bond disorder cannot produce a TAI as they lead only to a positive correction to *m*^22^,^23^. So far, however, the TAI phase was not found in the Kane-Mele model on a honeycomb lattice, describing for example graphene or proposed TIs such as silicene, germanene and stanene^24^,^25^,^26^,^27^. First indications to this phase were already found, showing that the Kane-Mele model without a staggered sublattice potential hosts extended bulk states even for large disorder strengths^28^.

In this paper we show the existence of TAIs in the Kane-Mele model by means of tight-binding calculations. The interplay between the parameters characterizing intrinsic spin-orbit coupling (SOC) *λ*_SO_, extrinsic Rashba SOC *λ*_*R*_, and a staggered sublattice potential *λ*_ν_ turns out to be crucial for the visibility of TAIs, and we calculate the parameter ranges in which TAIs can be observed. We find analytically that to lowest order in *W*, the parameters *λ*_SO_ and *λ*_*R*_ are not renormalized with increasing disorder strength, in contrast to *λ*_ν_. However, a new effective hopping *λ*_*R*3_ is generated due to the disorder, which is related but not identical to *λ*_*R*_. Although *λ*_*R*_ is not a crucial ingredient for the existence of TAIs, it significantly alters the physics of topological insulators in various ways^29^,^30^ and, as we will show below, strongly affects the TAI state.

Even though recently first signs of a TAI phase may have been found experimentally in evanescently coupled waveguides^31^, there has been no experimental evidence so far for the existence of the TAI phase in fermionic systems. The main difficulty is the requirement of a rather large and specific amount of disorder, which is difficult to control in the topological insulators currently investigated, where the 2D TI layer is buried inside a semiconductor structure. In contrast, producing and controlling disorder in 2D materials described by the Kane-Mele model could be much easier. Disorder in 2D materials with honeycomb structure can be produced by randomly placed adatoms^32^,^33^ or a judicious choice of substrate material^34^,^35^,^36^,^37^. Moreover, a sizeable staggered sublattice potential can be generated via a suitable substrate material^38^. Other means of engineering disorder were proposed in periodically driven systems^39^,^40^. Finally, honeycomb structures with the SOC necessary to produce a topological phase have already been realized using ultracold atoms in optical lattices^41^, in which disorder can in principle be engineered.

## Results

### Setup

The basis of our calculations is the Kane-Mele model^5^ given by the following Hamiltonian on a tight-binding honeycomb lattice


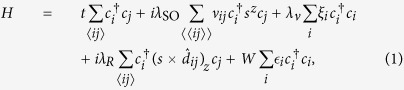


which has been supplemented by an on-site Anderson disorder term with disorder strength *W* and uniformly distributed random variables 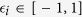. The summations over the lattice sites 〈*ij*〉 and 〈〈*i*, *j*〉〉 include all nearest neighbors and next-nearest neighbors, respectively. The operators 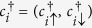, 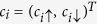 are creation and annihilation operators for the site *i* of the lattice. The parameters *t*, *λ*_SO_ and *λ*_*R*_ are the nearest-neighbor hopping strength, intrinsic SOC, and Rashba SOC, respectively. If the next-nearest neighbor hopping from site *j* to site *i* corresponds to a right-turn on the honeycomb lattice, then ν_*ij*_ = 1, otherwise ν_*ij*_ = −1. Furthermore, *s* =(*s*^*x*^, *s*^*y*^, *s*^*z*^) is the vector of Pauli matrices for the spin degree of freedom, and 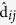 is the unit vector between sites *j* and *i*. The Wannier states at the two basis atoms of the honeycomb lattice are separated in energy by twice the staggered sublattice potential *λ*_ν_, with *ξ*_*i*_ = 1 for the *A* sublattice and *ξ*_*i*_ = −1 for the *B* sublattice. The lattice constant is *a*.

**Figure 1:**
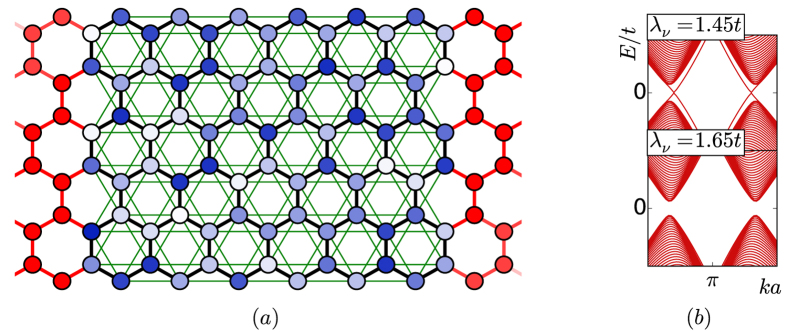
(**a**) Toy model illustrating the tight-binding terms in the Kane-Mele Hamiltonian (1). The blue color scale marks different on-site potentials. Thick black lines correspond to nearest-neighbor hopping and Rashba SOC, while thin green lines correspond to intrinsic SOC. The leads attached at both sides (red color) are modeled by a hexagonal lattice with nearest-neighbor hopping term and finite chemical potential. In this example, the sample has width *w* = 5*a* and length *l* = 6*a*. Much larger sample sizes of *w* = 93*a* and length *l* = 150*a* were used in the calculations described below. (**b**) Band structures of infinitely long samples of width *w* = 93*a* for two different values of *λ*_ν_ showing a topologically nontrivial and a trivial gap. Vertical and horizontal axis correspond to energy in units of *t* and dimensionless momentum, respectively. Parameters are *λ*_SO_ = 0.3*t* and *λ*_*R*_ = 0.

The band structure of this model depends strongly on the parameter set *λ*_SO_, *λ*_*R*_, and *λ*_ν_. In the clean limit and for *λ*_*R*_ = 0, the system will be a topological insulator for 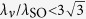 and a trivial insulator otherwise^5^. The tight-binding lattice and examples for the band structure in the clean limit are displayed in Fig. 1. For *W* = *λ*_ν_ = 0, the system will be a topological insulator if 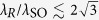 and a metal or semimetal otherwise. For finite *λ*_ν_ and *λ*_*R*_ the situation is more complex and a topological transition appears for values within these two boundaries.

### Numerical solution

**Figure 2:**
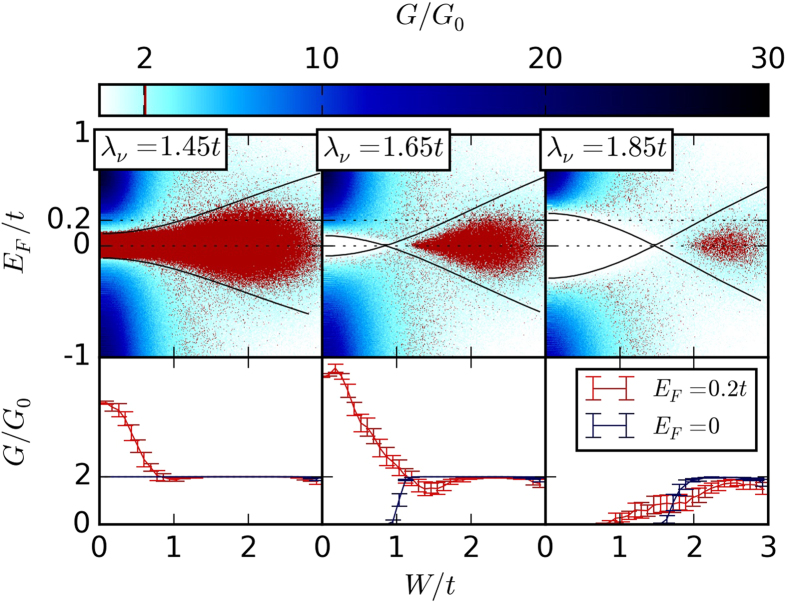
*Top row:* The conductance from the left to the right lead as a function of the disorder strength *W* (horizontal axis) and chemical potential *E*_*F*_ (vertical axis). The conductance varies from 0 (white) to 30*G*_0_ (dark blue). The quantized value of 2*G*_0_ (red for all conductances within [1.95*G*_0_, 2.05*G*_0_]) originates from two helical edge states. The three plots show the conductance for three different values of *λ*_ν_ that represent, respectively, a topological insulator, a TAI, and a TAI at the transition to an ordinary insulator. The black lines are obtained from a lowest-order Born approximation without any adjustable parameter. The two dotted lines mark the energies *E*_*F*_ = 0, *E*_*F*_ = 0.2*t*. *Bottom row:* The conductance at fixed chemical potentials *E*_*F*_ = 0 (black) and *E*_*F*_ = 0.2*t* (red) for the same parameters as in the top row. The errors bars originate from an averaging procedure over 100 disorder configurations. The vanishing error bars in the regions with a conductance of 2*G*_0_ show that the topological phase is stable irrespective of the exact disorder configuration. The system parameters are *w* = 93*a*, *l* = 150*a*, *λ*_SO_ = 0.3*t*, and *λ*_*R*_ = 0.

For *λ*_*R*_ = 0, we find a TAI phase for parameters close to the topological transition at 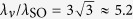. Changing this ratio corresponds to changing the band mass in the case of the BHZ model. Figure 2 shows the conductance for different values of *λ*_ν_. We find that for 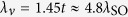 the system is a topological insulator. For *W* = 0, i.e., in the clean case, the conduction and valence bands are separated by a red region with a quantized conductance of 2*G*_0_. Remarkably, with increasing disorder strength, the states in the conduction and valence bands localize, but the helical edge states that are responsible for the conductance of 2*G*_0_ exist for an even larger energy window. The conductances and the vanishing error bars for the two distinct energy values *E*_*F*_ = 0, *E*_*F*_ = 0.2*t* in the lower row of Fig. 2 show that the conductance quantization, and with it the topological nature of the system, persist for the vast majority of microscopic disorder configurations. Interestingly, for *λ*_ν_ = 1.65*t* = 5.5*λ*_SO_, the system is a trivial insulator at *W* = 0. The trivial gap closes however, and at *W* ≈ *t* the system develops a topologically non-trivial gap and edge states. This can be seen from the quantized conductance. Finally, for *λ*_ν_ = 1.85*t* ≈ 6.2*λ*_SO_, the closing of the trivial gap and re-opening of the topological gap happens at a disorder strength which is strong enough to destabilize the emergent topological phase. Features of the conductance quantization can still be seen, but this behavior is not that robust anymore. As no averaging is done in the upper row of Fig. 2, and a new disorder configuration is taken for every data point, destabilization of the topological phase can be seen by red and white speckles in the figure.

**Figure 3:**
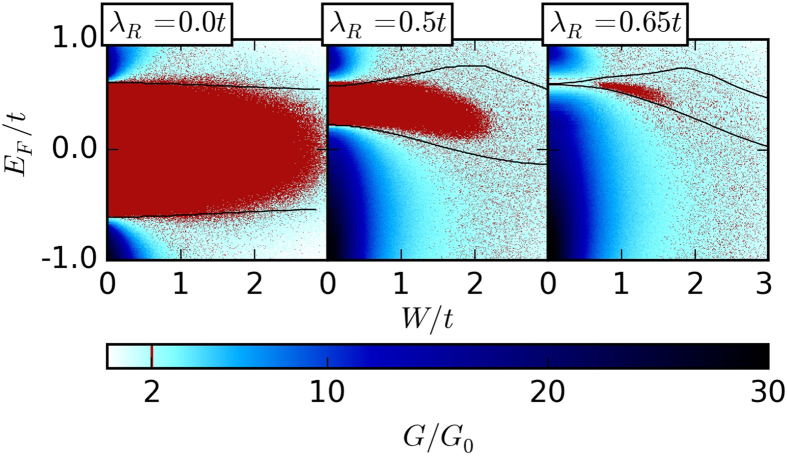
The conductance for increasing disorder strength *W* and chemical potentials *E*_*F*_ for three different values of *λ*_*R*_ = 0 (left), *λ*_*R*_ = 0.5*t* (middle) and *λ*_*R*_ = 0.65*t* (right). The system parameters are *w* = 93*a*, *l* = 150*a*, *λ*_SO_ = 0.3*t*, and *λ*_ν_ = 0.95*t*. The black lines are obtained from a lowest-order Born approximation without any fitting parameter. The conductance color code is the same as in Fig. 2.

We find that no TAI exists without staggered sublattice potential (*λ*_ν_ = 0). If both *λ*_ν_ and *λ*_*R*_ are finite, the TAI phase is in general less pronounced, see Fig. 3. The plot on the right shows the closing of a trivial gap and emergence of a topological phase at *W* ≈ 0.5*t*.

Furthermore, we observe that the simultaneous presence of intrinsic and Rashba SOC (both *λ*_*R*_ ≠ 0 and *λ*_*SO*_ ≠ 0) destroys the particle-hole symmetry in the spectrum. In the absence of Rashba SOC, the symmetry operator ϒ, which acts on the lattice operators as 
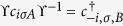
 and 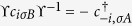 for the sublattices *A* and *B*, leaves the (disorder-free) Hamiltonian invariant. ϒ can be viewed as particle-hole conjugation combined with spatial inversion, and the inversion is needed to leave the staggered sublattice potential term invariant.

### Lowest-order Born approximation

In the self-consistent Born approximation, the self-energy ∑ for a finite disorder strength is given by the following integral equation^15^,^42^


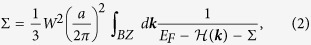


where 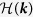 is the Fourier transform of *H* in the clean limit^5^. The coefficient 1/3 originates from the second moment 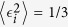 of the uniform distribution function of the disorder amplitudes, and *E*_*F*_ is the chemical potential. The integration is over the full first Brillouin zone. We use the lowest-order Born approximation, which means setting ∑ = 0 on the right-hand side of the equation.

After a low-energy expansion of 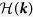, the integral can be evaluated analytically^15^ for *λ*_*R*_ = 0. This requires keeping the terms up to second order in ***k*** wherever this is the leading ***k***-dependent order. The evaluation yields the renormalized staggered sublattice potential


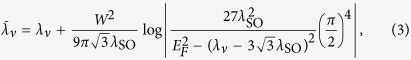


For a certain set of parameters, the logarithm can be negative and 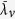 is reduced compared to *λ*_ν_. Moreover, we find that *λ*_SO_ is not renormalized to order *W*^2^. Therefore, it is possible to obtain 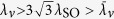. The system thus makes a transition from a trivial insulator to a topological insulator with increasing *W*.

For a more quantitative treatment, we evaluate the integral for the full Hamiltonian 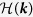 numerically. The self-energy ∑ is then written as a linear combination of several independent contributions


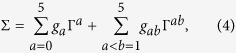


with 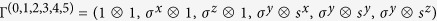 and Γ^*ab*^ = [Γ^*a*^, Γ^*b*^]/(2*i*). Here, *σ*^*x*^, *σ*^*y*^, *σ*^*z*^ denote the Pauli matrices for the sublattice index. This leads to the following equations for the renormalized quantities


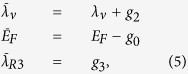


whereas 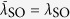 and 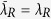 remain unrenormalized to lowest order in *W*. Surprisingly, a new coupling 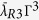 is created by the disorder. This coupling has the matrix structure Γ^3^, which is similar but not identical to the one for Rashba SOC. Expressing this new term in the lattice coordinates of Eq. (1) reveals that it corresponds to a Rashba-type nearest-neighbor hopping term which is asymmetric and appears only for bonds that are parallel to the unit vector (0, 1),


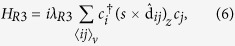


where 〈*ij*〉_ν_ stands for summations over strictly vertical bonds only. Furthermore, we find to lowest order in *W* that 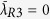 for *λ*_*R*_ = 0.

For *W* = *λ*_*R*_ = 0, the upper and lower edge of the gap are at the energies 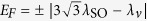. This is the case for both topological and trivial insulators. Extrapolation of these equations to finite *W* leads to the conditions 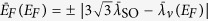. The solid black lines in Fig. 2 are the two solutions to these equations and describe the closing and reopening of the gap qualitatively for small *W*.

For finite *λ*_*R*_ and therefore finite *λ*_*R*3_, there is no analytical expression of the gap energy. In this case, we read off the positions of the gap edges from band structure calculations for several values of *λ*_*R*_ and *λ*_*R*3_. An interpolation leads to two functions *h*_*U*,*L*_(*λ*_ν_, *λ*_*R*_, *λ*_*R*3_) for the upper and lower band edge in the clean system. Replacing the unperturbed by the renormalized parameters yields two equations


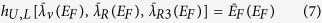


The solutions of these equations are indicated by the solid black lines in Fig. 3. Hence, these results agree with the numerical data for small *W* without any fitting parameter. Deviations appear for larger *W*, when the lowest-order Born approximation is not applicable.

### Phase diagram

**Figure 4:**
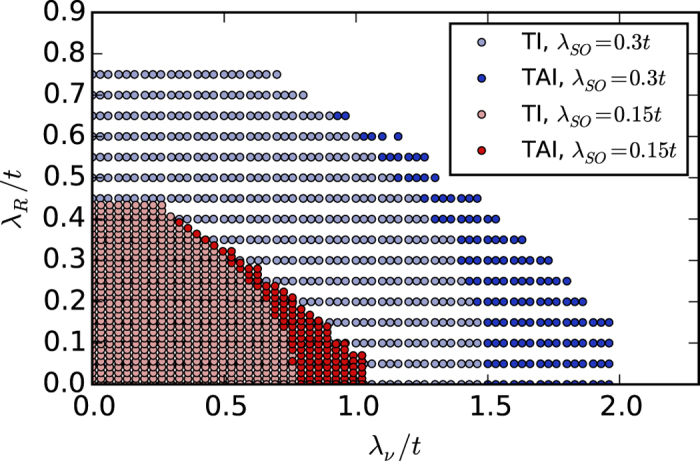
Phase diagram in the (*λ*_*ν*_, *λ*_*R*_) plane. Strong blue (red) color marks the region for which a TAI exists for *λ*_SO_ = 0.3*t* (0.15*t*). Transparent blue (red) color indicates the regions where a topological insulator is found for zero disorder. Each dot represents an individual simulation of the kind illustrated in Fig. 2.

**Figure 5:**
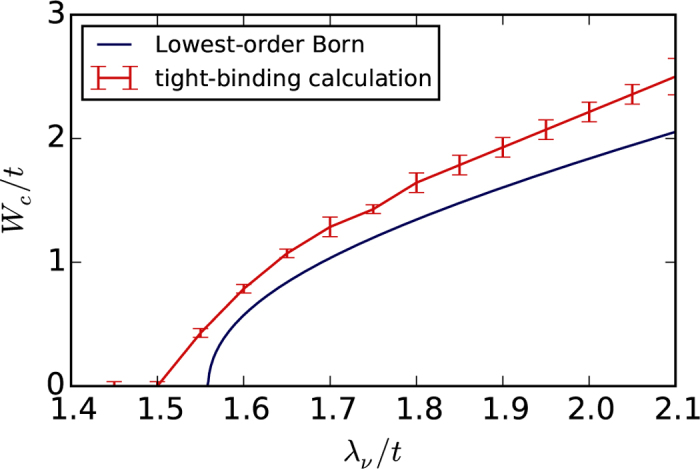
Critical value of the disorder strength for the TAI transition along the line *λ*_*R*_ = 0 for *λ*_SO_ = 0.3*t*. Comparison between tight-binding simulation and analytical results.

Figure 4 shows a phase diagram as a function of *λ*_ν_ and *λ*_*R*_ based on the tight-binding simulations. The dark color marks the regions for which a critical disorder strength *W*_*c*_ exists above which the system is a TAI (blue for *λ*_SO_ = 0.3*t*, red for *λ*_SO_ = 0.15*t*). The TAI phase is located along the boundary separating trivial from topological insulators in the clean case. Towards larger *λ*_*R*_, the TAI region becomes narrower and eventually vanishes above a critical *λ*_*R*_. Figure 5 shows the critical disorder strength *W*_*c*_ as a function of *λ*_ν_ for a fixed value of *λ*_*R*_.

In Figs 2 and 3 rather large values of the parameters *λ*_SO_, *λ*_ν_ and *λ*_*R*_ were chosen to better visualize the effect. The TAI phenomenon scales down also to smaller values of the parameters, as the red region in the Fig. 4 indicates, but the TAI phase becomes less pronounced in the conductance plots and is harder to identify. Material parameters for stanene for example are *t* = 1.3 eV, *λ*_SO_ = 0.1 eV^43^ and *λ*_*R*_ = 10 meV^44^. We suspect that disorder, e.g., originating from missing or dislocated atoms, can reach disorder strengths in the eV range.

### Alternative disorder models

Anderson disorder is a special model for disorder which is not necessarily representative for all TI materials. To better understand the effect of the disorder model, we briefly remark on the following disorder Hamiltonian


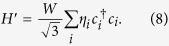


In contrast to the Anderson disorder model, where a random potential is assigned to *every* lattice site, here the distribution function for *η*_*i*_ is such that only a fraction 0 < *ρ* ≤ 1 of the sites are affected by disorder. Denoting the total number of sites by *N*, we assume *η*_*i*_ = 1 on *ρN*/2 sites, *η*_*i*_ = −1 on *ρN*/2 sites, and *η*_*i*_ = 0 on the remaining sites. The disorder amplitude *W* is constant. Because 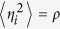, the normalization factor in Eq. (8) ensures that the mean squared disorder strength is equal to the Anderson disorder case for *ρ* = 1.

For general *ρ*, the prefactor in Eq. (2) is thus replaced by *ρW*^2^/3. The lowest-order Born approximation for the disorder model (8) therefore predicts that a reduced disorder density *ρ* can be exactly compensated by an increased amplitude *W*. For large enough *ρ*, this is indeed confirmed in the tight-binding simulations.

However, because a single impurity (*ρ* = 1/*N*) cannot destroy the topological phase, it is clear that the TAI phase should eventually vanish for *ρ* → 0 at arbitrary *W*. Nevertheless, we find numerical evidence for the TAI phase at surprisingly low impurity densities. A TAI region remains pronounced for densities as low as *ρ* = 0.1.

## Discussion

In conclusion, we have shown that the topological Anderson insulator is a significantly more universal phenomenon than previously thought. Using a combination of an analytical approach and tight-binding simulations, we have established that the topological Anderson insulator appears in the Kane-Mele model that describes potential topological insulators such as silicene, germanene, and stanene and that can also be realized in optical lattices. We have observed a transition from a trivially insulating phase to a topological phase at a finite disorder strength and have mapped out the phase diagram as a function of the staggered sublattice potential (~*λ*_ν_) and the Rashba spin-orbit coupling (~*λ*_*R*_). The new Anderson insulator exists at the boundary between trivial and topological insulators for small *λ*_*R*_ and finite *λ*_ν_, but not at the boundary between a semimetal and a topological insulator for small *λ*_ν_ and finite *λ*_R_. Since the Kane-Mele model on a honeycomb lattice describes a wide class of candidate materials for topological insulators, we hope that our work will trigger experimental efforts to confirm the existence of the topological Anderson insulator.

## Methods

The numerical simulations were done with the tight-binding Hamiltonian (1) on a honeycomb lattice with rectangular shape of width *w* = 93*a* and length *l* = 150*a* using the Kwant code^45^. A smaller version of the sample is shown in Fig. 1. Both the upper and lower edge are taken to be of zigzag type. At the left and right edges two semi-infinite, metallic leads of width *w* are attached. The leads are also modeled by a honeycomb lattice with only nearest-neighbor hopping and a finite on-site energy of 1.2*t* to bring them into the metallic regime.

## Additional Information

**How to cite this article**: Orth, C. P. *et al.* The topological Anderson insulator phase in the Kane-Mele model. *Sci. Rep.* **6**, 24007; doi: 10.1038/srep24007 (2016).
